# Temperature Gradient Characteristics of Rubber-Modified Asphalt Pavement Under Dramatic Cooling–Heating Cycles

**DOI:** 10.3390/ma17235754

**Published:** 2024-11-24

**Authors:** Meiyan Huang, Jianguo Wei, Ping Li, Yuming Zhou, Yiliang Li, Wenju Peng, Xuan Xiao

**Affiliations:** 1 School of Traffic and Transportation Engineering, Changsha University of Science & Technology, Changsha 410114, China; jianguowei9969@126.com (J.W.); li_ping@csust.edu.cn (P.L.); yuming_zhou4092023@163.com (Y.Z.); li_yiliang@126.com (Y.L.); pwj1112@foxmail.com (W.P.); xiao990601@163.com (X.X.); 2 National Engineering Research Center of Highway Maintenance Technology, Changsha University of Science & Technology, Changsha 410114, China

**Keywords:** dramatic cooling–heating cycles, damage depth threshold, cyclic temperature changes, pavement temperature field

## Abstract

The periodic changes in climatic factors cause the pavement temperature field to change significantly, resulting in fatigue damage to the pavement caused by temperature stress, and the influence depth has a critical value. To reveal the influence range and variation pattern of the rubber-modified pavement temperature field under frequent rainfall and high temperatures, based on indoor tests and the finite element model, the evolution law of different influencing factors and pavement temperature fields was determined by a single factor sensitivity analysis method. The degree of influence of each influencing factor on the pavement temperature field was analyzed using the Pearson correlation. The results showed that with different asphalt mixture initial temperatures, the road surface temperature decreased from 20 °C to 40 °C under sudden rainfall. Repeated rainfall following high temperatures induces cyclic temperature changes 30 mm below the road surface. The pavement temperature difference increased linearly with the dramatic temperature difference, and the changes in the pavement temperature field were small when the number of cycles exceeded 30. The number of cycles and cycle temperature difference were the main factors affecting the changes in the pavement temperature field under dramatic cooling–heating cycles.

## 1. Introduction

With the global trend of extreme climate, conditions such as high temperatures, heavy rains, and severe cold have caused severe damage to infrastructure, especially roads. In particular, asphalt pavements are prone to early problems, such as cracking and rutting, under extreme temperature fluctuations [1,2,3]. In the southern coastal areas of China, including Guangdong, Fujian, Hainan, and Guangxi, rubber-modified asphalt is mostly used as the surface layer to resist continuous high temperatures [4,5]. Moreover, heavy rains are frequent in summer in these areas. The asphalt pavement temperature before rain can exceed 70 °C, and heavy rainfall can rapidly decrease to below 40 °C, resulting in rapid temperature decrements of up to 40 °C. Asphalt pavements are rapidly heated and dried between rainstorms, and repeated rainstorms put the asphalt mixture under dry–wet cycles of rainfall cooling and solar heating, thus forming dramatic cooling–heating cycles [6,7,8]. Frequent high temperatures and rainstorms cause dramatic changes in the pavement temperature field, with significant temperature differences on the pavement surface over a short period of time. Meanwhile, rainwater seeps to different depths of the pavement, facilitating heat convection with the pavement structure, thus exhibiting unique temperature field characteristics of the pavement under dramatic cooling–heating cycles. The pavement temperature field characteristics differ from those of the conventional temperature field. The interval between high temperature and rainfall is short, the variation range of the pavement temperature field is limited, and the influence depth of the pavement has a critical value. Therefore, the damage depth of the pavement under the influence of a large humidity–temperature cycle can be obtained, which has guiding significance for pavement design and maintenance in this area.

Scholars have conducted extensive research on the effect of external environmental changes on pavement temperature. Mohseni et al. [9] found that the maximum and minimum temperatures at different depths beneath the pavement changed linearly with surface and atmospheric temperatures. Through field measurements and finite element simulation, Chung et al. [10] analyzed and predicted the temperature gradients at different depths of asphalt pavements in different seasons. Chao et al. [11] employed the least squares method to quantify the relationship between environmental factors and pavement temperature and established a statistical model for predicting asphalt pavement temperature based on environmental data. Using the finite element method, Zhao et al. [12,13] analyzed the temperature field changes of asphalt pavements under large temperature differences and high temperatures in southern Xinjiang. With a deeper beneath-the-pavement surface, the effects of atmospheric temperature were significantly weakened, indicating a time lag in the effects of atmospheric temperature on the temperature of the pavement structure. Li et al. [14] built a prediction model for the temperature of the asphalt layer with a thickness of over 30 cm, encompassing factors like atmospheric temperature, solar radiation, and ground temperature. Luo et al. [15] found that the atmospheric temperature mainly affects the surface layer temperature field of the asphalt pavement, with small temperature field changes beyond a certain depth below the pavement surface. The maximum temperature difference of the underlying pavement structure exhibits hysteresis compared to that of the upper structure. Zhang et al. [16] analyzed the effects of continuous temperature changes on the temperature field of the asphalt pavement in ABAQUS, concluding that the maximum temperature and temperature increase rate of the asphalt pavement gradually decreased with the increased depth. However, the depth threshold has not yet been determined. The above research shows that pavement temperature is significantly affected by external environmental factors, and the effects of external temperature changes on the temperature field weaken with increasing pavement depth.

A further review found that current studies on the pavement temperature field mainly focus on climate and temperature changes over a long period of time [14,17,18,19,20], such as cyclic changes in different seasons, dates, and hours. However, few studies have focused on the temperature field under dramatic changes in external environmental factors over short periods of time, such as approximately ten minutes or even a few minutes. For example, the pavement temperature field gradient characteristics under the coupling effects of large temperature difference, high temperature, and water and the resultant fatigue damage depth threshold of the asphalt pavement. This study simulates the gradient characteristics of the pavement temperature field in high-temperature and rain-prone areas based on the dramatic cooling–heating cycle equipment independently developed by our research team. A rainwater layer was simulated based on the heat transfer theory and ABAQUS to analyze the variation pattern of the pavement temperature field under the coupling effect of the large temperature difference and rainwater, and to predict the long-term variation of the rubber-modified pavement temperature field under dramatic cooling–heating cycles. Correlation analyses were performed on the various factors influencing the temperature field.

## 2. Materials and Methods

### 2.1. Asphalt

The asphalt used included rubber-modified asphalt produced by Guangxi Transportation Science and Technology Group Co., Ltd. (Nanning, China) for the upper layer of the road surface and SBS-modified asphalt produced by Sinopec for the middle layer of the road surface. The relevant technical specifications of the asphalt are presented in Table 1 and Table 2.

### 2.2. Asphalt Mixture

In the laboratory tests, two Marshall specimens were used as the upper and lower layers to simulate the upper and middle parts of the pavement surface. The upper-layer asphalt mixture was AC-13 and the lower-layer asphalt mixture was AC-20. The asphalt mixture grading design is shown in Table 3. Through Marshall tests, the asphalt contents of AC-13 were determined to be 4.7%, and the asphalt content of AC-20 was determined to be 5%, respectively. The aggregate and mineral powders were composed of limestone. The performance indices are presented in Table 4 and Table 5.

### 2.3. Dramatic Cooling–Heating Cycle Test

**Figure 1:**
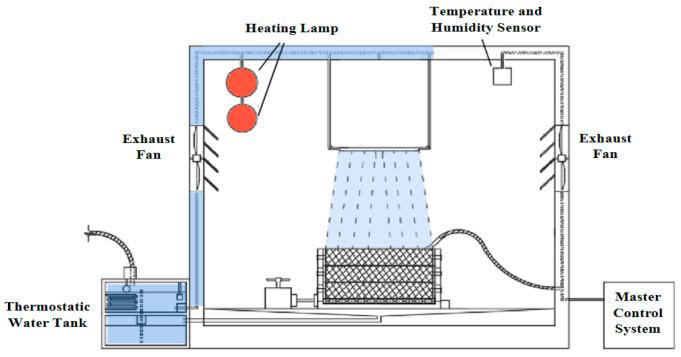
The principle and internal details of the dramatic cooling–heating cycle simulation test equipment.

A dramatic cooling–heating cycle simulation test equipment developed by our research team was used. As shown in Figure 1, it includes a heating system, rainfall system, water supply system, and dehumidification system. The heating system comprised six 275 W heating lamps and various temperature sensors in the box. The rainfall system mainly included square rain sprinklers, water temperature sensors, an adjustable flow rate pump, and a water-discharging test stand. The water supply system includes a thermostatic water tank, a water storage tank, a water level sensor, and a water pipe. To simulate the rapid decrease in temperature due to sudden rainfall on the high-temperature pavement in summer, the difference between the initial temperature of the asphalt mixture and the rain temperature was used to characterize the large temperature decreases of the pavement. To simulate the rapid heating and drying of the pavement after heavy rainfall, the rain duration was used to characterize the influence of the rain intensity on the pavement temperature. Meanwhile, high temperatures and rainstorms are often cyclical in summer, and repeated heating following rainstorms puts the asphalt mixture under dry–wet cycles of rainfall cooling and solar heating. These cycles exert cyclical effects on the pavement temperature. Therefore, different test parameters, such as the initial temperature of the asphalt mixture, rain temperature, rain duration, and number of cycles, were set to analyze the variation patterns of the pavement temperature field under dramatic cooling–heating cycles. In this test, the heating rate of the dramatic cooling–heating cycle equipment was set to 65.9 °C/h, the rain rate was set to 3.5 mm/min, and the rain temperature was 20 °C.

## 3. Model and Theory of Asphalt Mixture Temperature Field Under Dramatic Cooling–Heating Cycles

### 3.1. Pavement Heat Transfer Theory

#### 3.1.1. Pavement Heat Transfer Mode

**Figure 2:**
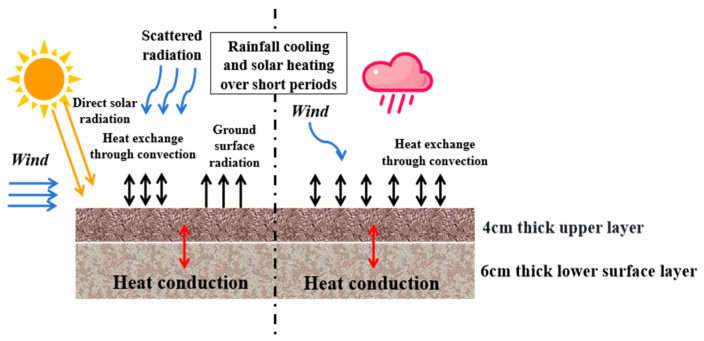
Schematic diagram of the pavement temperature field under dramatic cooling–heating cycles.

The temperature field of the pavement structure is mainly formed by heat conduction, heat radiation, and convection exchange [23]. The pavement surface undergoes heat exchange with the external environment through heat radiation and heat convection, and the interior of the pavement undergoes heat exchange through heat conduction. Figure 2 shows the analysis of the interaction between the external environment and the pavement structure.

(1) Heat radiation

Coastal high-temperature areas in China often have sudden rains following high temperatures and high temperatures again following rainstorms. The pavement’s temperature increase is mainly due to the solar radiation it absorbs between the rainfall in the cooling–heating cycles. Equation (1) expresses its relationship with the duration of continuous high temperatures. The road surface radiation is related to the surface temperature, as described in Equation (2).

(1) Q=Q(t)

(2) $$q_{s}=\alpha •\delta •T_{s}^{4}$$

where Q is the solar radiation between the rainfall in the cooling–heating cycles; t is the time between rainfall in the cooling–heating cycles; $q_{s}$ is the road surface radiation; α is the road surface radiation coefficient; δ is the Stefan–Boltzmann constant $\left(5.68\times 10^{-8}/W\cdot {\left(m^{2}\cdot k^{4}\right)}^{-1}\right)$; and $T_{s}$ is the road surface temperature (K).

(2) Convection exchange

The heat exchange between the pavement surface and the external environment follows Newton’s Law of Cooling, i.e., the heat dissipation rate of an object is proportional to the temperature difference between the object and the surrounding environment, as expressed in Equation (3).

(3) $$q_{c}=\omega \cdot (T_{s}-T_{a})$$

where ω is a constant, which is the convection conversion coefficient related to the wind speed $\left(W\cdot {\left(m^{2}\cdot k^{4}\right)}^{-1}\right)$; and $T_{a}$ is the atmospheric temperature (k).

(3) Heat conduction

Heat conduction is the transfer of heat from high-temperature areas to low-temperature areas within an object or from a high-temperature object to a low-temperature object. The heat conductivity equation for isotropic materials with the same heat conductivity can be derived according to the Fourier thermal conductivity law, as expressed in Equation (4).

(4) $$q_{x}=-\lambda \frac{\partial T}{\partial x}$$

where $q_{x}$ is the heat flux density, the heat conducted per unit area along the X axis (W/m^2^); λ is the heat conductivity of the material (W/(m·K)); and $\frac{\partial T}{\partial X}$ is the gradient of the temperature function along the X axis (k/m).

#### 3.1.2. Transient Nonlinear Heat Transfer

With dramatic cooling–heating cycles, the asphalt pavement undergoes rainfall cooling and solar heating cycles, leading to transient temperature field changes in the asphalt mixture. During this process, the internal temperature, heat conduction, and boundary conditions of the pavement change significantly with time. According to the energy conservation theorem, the transient heat balance of the asphalt mixture can be expressed in the form of a matrix Equation (5).

(5) $$\left[C\right]\left\{T^{\prime }\right\}+\left[\varepsilon \right]\left\{T\right\}=\left\{Q\right\}$$

where $\left[C\right]$ is the specific heat capacity matrix; $\left[\varepsilon \right]$ is a matrix of heat conductivity, convection coefficient, and radiation coefficient; $\left\{T^{\prime }\right\}$ is the derivative of temperature versus time; $\left\{T\right\}$ is the node temperature vector; $\left\{Q\right\}$ is the node heat flux vector.

The thermal properties of asphalt pavement are nonlinearly related to temperature, density, and boundary conditions. The heat balance equation for nonlinear thermal energy analysis is expressed in Equation (6).

(6) $$\left[C(T)\right]\left\{T^{\prime }\right\}+\left[\varepsilon (T)\right]\left\{T\right\}=\left\{Q(T)\right\}$$

#### 3.1.3. Heat Conduction Differential Equation

Asphalt mixtures are isotropic materials with known thermophysical parameters. It is assumed that there is a heat source inside the asphalt mixture, with Φ representing the heat energy generated or consumed per unit volume per unit time (W/m^3^; positive for heat generation and negative for heat consumption). It is also assumed that the thermophysical parameters of the thermally conductive object are a function of temperature.

**Figure 3:**
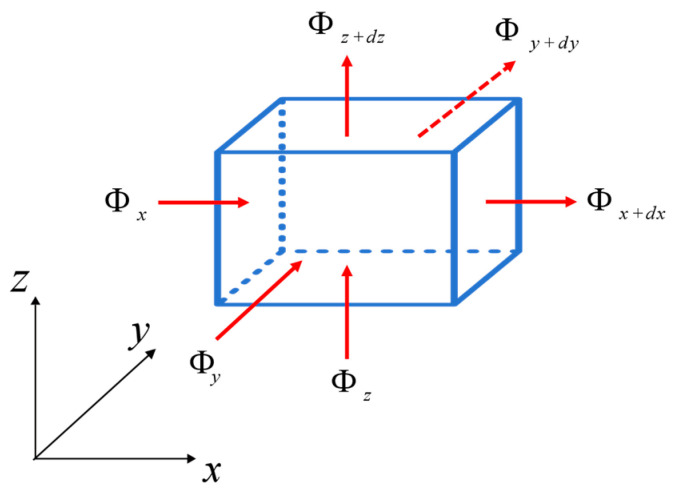
Microelement heat conduction heat balance analysis.

A microelement parallel hexahedron can be randomly selected from the thermally conductive object for energy balance analysis. A heat flow in any direction can be decomposed into components along the x, y, and z axes, represented as $\Phi_{x^{\prime }}$, $\Phi_{y^{\prime }}$ and $\Phi_{z}$ in Figure 3. According to Fourier’s law, the heat flux of different microelement surfaces can be expressed in Equation (7).

(7) $$\left.\begin{array}{l} \Phi_{x}=-\lambda \frac{\partial t}{\partial x}dydz\\ \Phi_{y}=-\lambda \frac{\partial t}{\partial y}dxdz\\ \Phi_{z}=-\lambda \frac{\partial t}{\partial z}dxdy \end{array}\right\}$$

where ${\left(\Phi_{x}\right)}_{x}$ represents the value of the x direction component $\Phi_{x}$ of the heat flow at point x, and the rest can be derived by analogy. The heat flux of the microelement derived based on surfaces x=x+dx, y=y+dy and z=z+dz can also be expressed in Equation (8) according to Fourier’s law.

(8) $$\left.\begin{array}{l} \Phi_{x}=\Phi_{x}+\frac{\partial \Phi_{x}}{\partial x}dx=\frac{\partial }{\partial x}\left[-\lambda (\frac{\partial t}{\partial x}dydz\right]dx\\ \Phi_{y}=\Phi_{y}+\frac{\partial \Phi_{y}}{\partial y}dy=\frac{\partial }{\partial y}\left[-\lambda (\frac{\partial t}{\partial y}dxdz\right]dy\\ \Phi_{z}=\Phi_{z}+\frac{\partial \Phi_{z}}{\partial z}dz=\frac{\partial }{\partial z}\left[-\lambda (\frac{\partial t}{\partial z}dxdy\right]dz \end{array}\right\}$$

### 3.2. Basic Assumptions and Boundary Conditions

#### 3.2.1. Basic Assumptions

The following assumptions are made for the pavement structure under dramatic cooling–heating cycles: (1) each structural layer of the road surface is uniform, continuous, and isotropic; (2) the heat conduction between the structural layers is continuous; and (3) considering that the rainwater only directly contacts the surface of the pavement during rainfall, the transient temperature changes inside and on the sides of the pavement are relatively small, and the sides of the Marshall specimen are set as adiabatic boundaries. (4) The temperatures of the lower surface layer and below were set to a constant value.

#### 3.2.2. Boundary Conditions

With rainfall following high temperatures, the road surface temperature decreases rapidly, with heat exchange between rainwater and the road surface through convection. Between rainfall events, the road surface temperature rises due to solar radiation, and the pavement transfers its internal heat and the heat absorbed from the atmosphere to the middle surface layer through heat conduction. This cycle repeats. The heat transfer boundary conditions at the different stages were as follows:

Rainfall cooling stage:

(9) $$-\lambda \frac{\partial T_{s}}{\partial y}=\omega •(T_{w}-T_{s})$$

Solar heating stage:

(10) $$\lambda \frac{\partial T_{upper}}{\partial y}=\lambda \frac{\partial T_{middle}}{\partial y}$$

where $T_{w}$ is the rain temperature; $T_{upper}$ is the upper surface layer temperature; $T_{middle}$ is the middle surface layer temperature; y is the coordinate in the pavement depth direction.

## 4. Finite Element Calculation Model of the Temperature Field Under Dramatic Cooling–Heating Cycles

### 4.1. Finite Element Modeling

**Figure 4:**
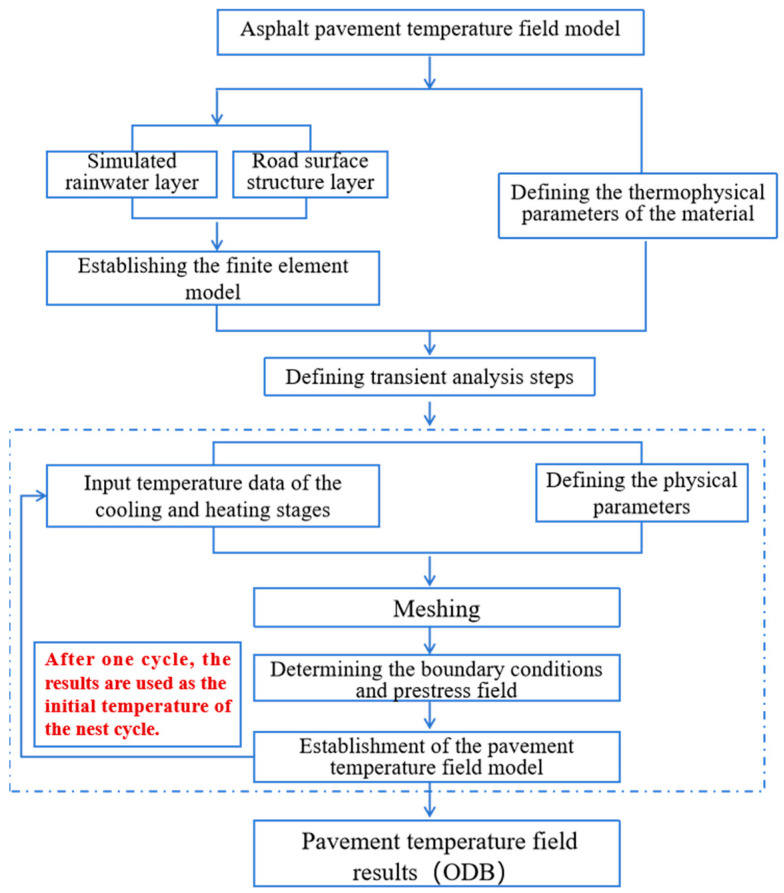
Modeling process of the temperature field under dramatic cooling–heating cycles.

**Figure 5:**
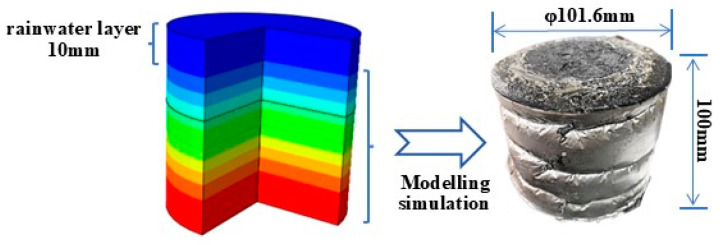
Two-dimensional axial symmetric model of the pavement temperature field.

The finite element model of the pavement temperature field under dramatic cooling–heating cycles was established in ABAQUS 2021. In order to match the specimen for temperature field measurements in the laboratory, a model was constructed with two-dimensional axial symmetry. The model was 101.6 mm wide and 110 mm high (100 mm for the Marshall specimen and 10 mm for the rainwater layer). The modeling process is presented in Figure 4 and Figure 5.

### 4.2. Model Validation and Efficacy Analysis

#### 4.2.1. Finite Element Model Validation

**Figure 6:**
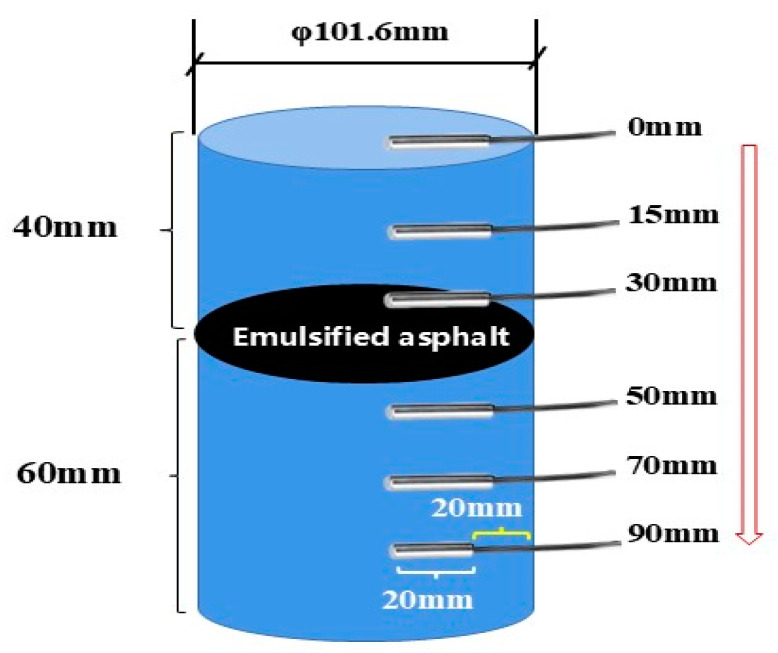
Schematic diagram of the layered Marshall specimen and its preparation.

**Figure 7:**
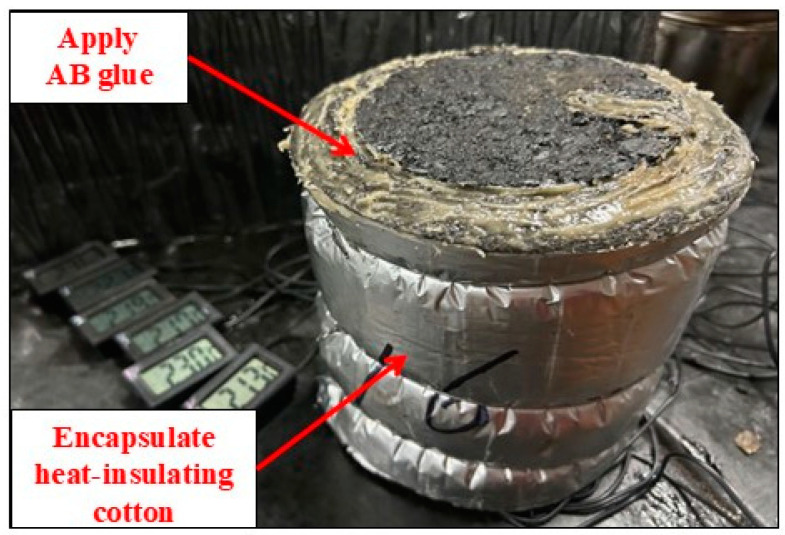
Marshall specimen waterproofing and thermal insulation.

To validate the finite element simulation model, a layered Marshall specimen was prepared according to the T0702-2011 Chinese standard [24]. Temperature sensors were installed at 4 mm, 15 mm, 30 mm, 50 mm, 70 mm, and 90 mm below the road surface to measure the road surface temperature field in the laboratory (Figure 6). Considering that only the asphalt pavement surface is in direct contact with rainwater during rainfall, the sides of the specimen were wrapped with waterproof and heat-insulating insulation cotton, and the edges of the insulation cotton were sealed to the specimen using AB glue, thus rendering the layered Marshall specimen waterproof and thermal insulated (Figure 7).

**Figure 8:**
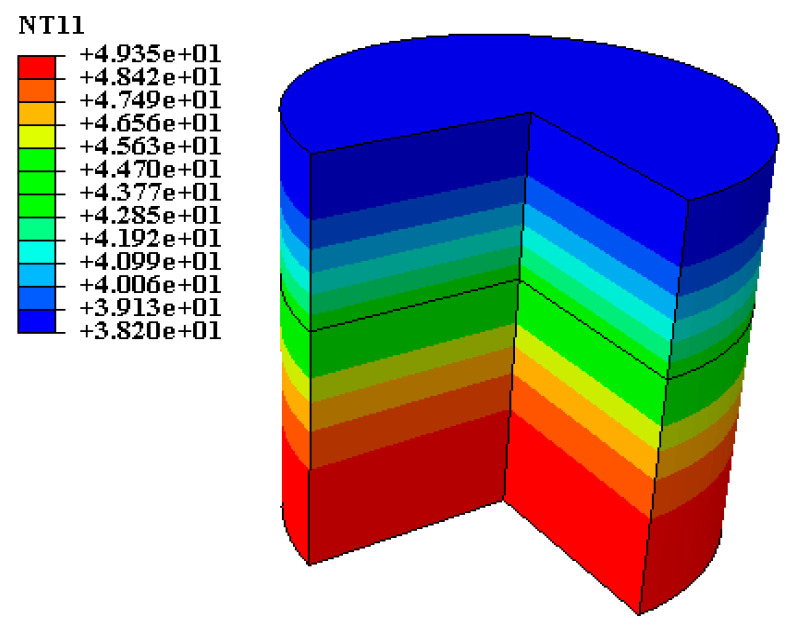
Pavement temperature field cloud diagram.

The validity of the finite element model was verified using the measured temperature field data in the laboratory. The temperature field was constructed according to the boundary conditions. The initial temperature of the rainwater layer and the field temperature were set to 20 °C, the initial temperature of the asphalt mixture specimen was set to 50 °C, the time step was set to 1 min during rainfall and 2 min during heating, and the number of cycles was set to 5. The simulation results are presented in Figure 8.

To quantitatively analyze the validity of the finite element model, three error indicators, i.e., maximum deviation δ, mean absolute error (MAE), and root mean square error (RMSE), were calculated to measure the degree of deviation between the simulated values and the measured values [10]. The relevant calculation process is as follows.

(11) $$\delta =|T_{i}-T_{j}|$$

(12) $$MAE=\frac{\sum_{1}^{n}\left|T_{i}-T_{j}\right|}{n}$$

(13) $$RMSE=\sqrt{\frac{1}{n}\sum_{i=1}^{n}(T_{i}-T_{j}{)}^{2}}$$

where *T_i_* and *T_j_* represent the measured and simulated temperatures, respectively, and *n* is the number of test specimens. Smaller δ, MAE, and RMSE values indicate smaller errors between the measured and simulated values and a higher model simulation accuracy.

#### 4.2.2. Analyses of Simulated and Measured Temperatures 4 mm Beneath the Road Surface

**Figure 9:**
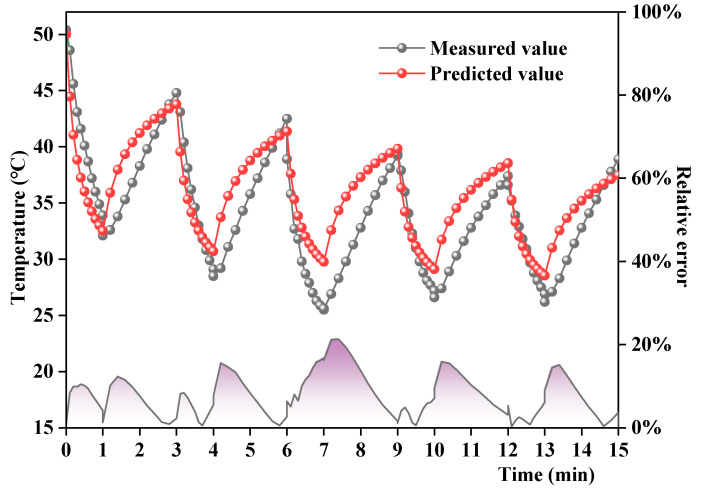
Simulated and measured temperatures 4 mm beneath the road surface.

The simulated and measured temperatures 4 mm beneath the road surface are presented in Figure 9. The simulated and measured temperatures 4 mm beneath the road surface were roughly consistent. The maximum δ value was 21.7% at the final heating time point of the fifth cycle. The **δ** values at other test time points were within 20%. Thus, the finite element model can effectively simulate the pavement temperature field under repeated rainfall over short periods with high reliability.

#### 4.2.3. Analyses of Simulated and Measured Temperatures at Different Depths Beneath the Pavement Surface

**Figure 10:**
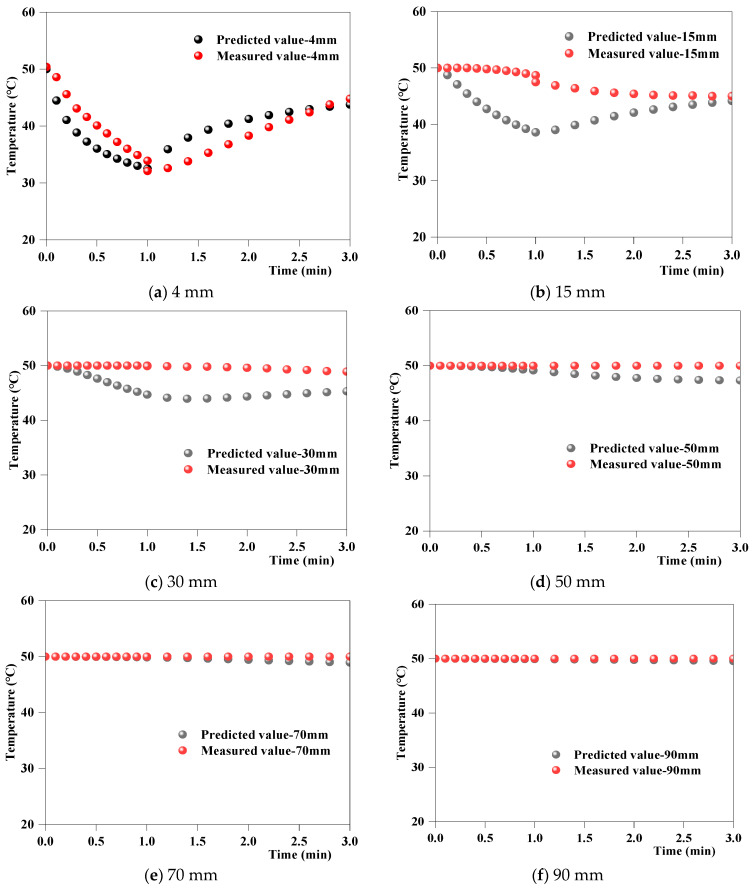
Simulated and measured temperature fields at different depths beneath the pavement surface.

Figure 10 shows the changes in the simulated and measured temperatures over time at different depths beneath the road surface during the first cycle. The calculated error indicators are listed in Table 6. The errors at 15 mm beneath the road surface were relatively large, with a maximum relative error δ of only 13.56%, an MAE of 2.54, and an RMSE of 3.01. In contrast, the errors at the other depths were small. Thus, the deviation between the simulated and measured temperatures at different depths beneath the road surface was small, and the model prediction accuracy was good.

The relationship between the simulated and measured temperature fields at different depths under the road surface was further analyzed, and the error indicators were calculated (Table 6). The errors at 15 mm beneath the road surface were relatively large, with a maximum relative error δ of 10.1%, MAE of 5.08, and RMSE of 6.02. In contrast, the errors at the other depths were small. Thus, the deviation between the simulated and measured temperatures at different depths beneath the road surface was small, and the prediction accuracy of the finite element model was good.

### 4.3. Evolution Pattern Analysis of the Pavement Temperature Field Under Dramatic Cooling–Heating Cycles

The pavement temperature field is mainly affected by the thermophysical properties of the asphalt mixture (internal factors), such as heat conductivity and specific heat, and external factors like atmospheric temperature, solar radiation, wind speed, sunshine duration, and rainfall. Under dramatic cooling–heating cycles, the difference between the initial temperature of the asphalt mixture and the rain temperature, rain duration, and the number of cycles significantly affected the pavement temperature field. To clarify the variation pattern of the pavement temperature field, a single variable sensitivity analysis was performed to determine the degree of influence of each factor. That is, only one influencing factor is analyzed at a time, with the other factors fixed. The relevant fixed reference values are listed in Table 7 [25,26,27,28,29]. To facilitate the subsequent experimental analysis, conditions with different influencing factors were uniformly encoded. For example, the condition with an initial temperature of the asphalt mixture of 70 °C, rain temperature of 20 °C, rain duration of 1 min, and heating time of 2 min was encoded as 70-20R-1J-2S. The other conditions are encoded likewise.

#### 4.3.1. Impact of the Initial Temperature

(1) Effect of initial temperature on road surface temperature

**Figure 11:**
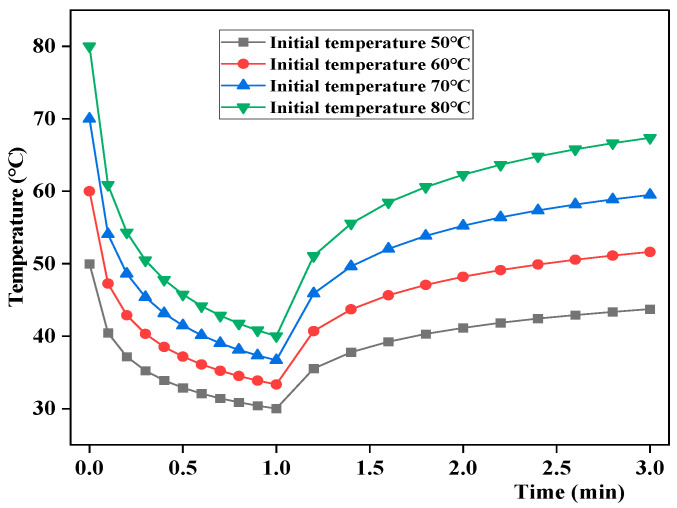
Effect of initial temperature on road surface temperature.

In summer, the pavement temperature rises continuously due to high atmospheric temperatures and decreases rapidly due to rainfall, and the resulting large temperature difference significantly impacts the pavement performance. The initial temperature of the asphalt mixture was set to 50 °C, 60 °C, 70 °C, and 80 °C to study its effect on the pavement temperature field. The results are shown in Figure 11.

**Figure 12:**
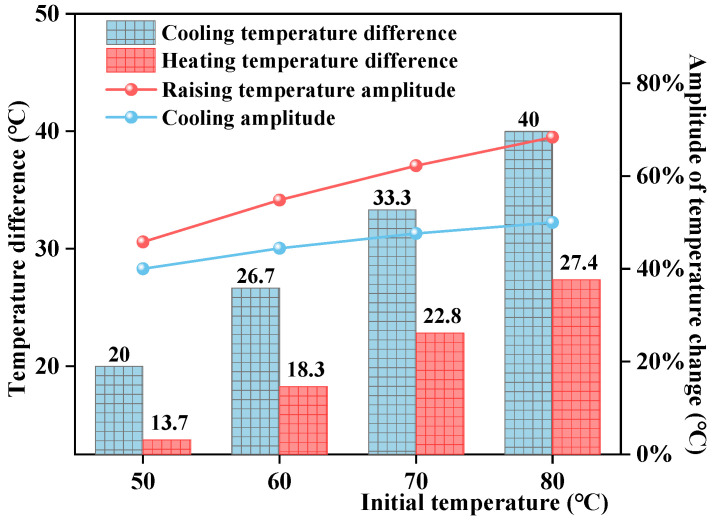
Temperature changes during cooling and heating of the road surface at different initial temperatures.

As shown in Figure 11, the road surface temperature first decreased and then increased at different initial asphalt mixture temperatures. At a higher initial temperature, the temperature decrements and increments during cooling and heating were greater. With an initial temperature of 50 °C, the cooling temperature decrement was 20.0 °C, while the heating temperature increment was 13.7 °C. At an initial temperature of 80 °C, the temperature decrement and increment during cooling and heating are 40.0 °C and 27.4 °C, respectively. The temperature change amplitudes of the cooling and heating stages were used to further quantify the influence of different initial temperatures on the evolution of road surface temperature. The results are shown in Figure 12. As the initial temperature increased, the temperature change amplitudes of the cooling and heating stages increased, and the temperature change gradient during heating was greater than that during cooling. These results indicate that with a higher pavement temperature, the surface temperature variation was greater, and the temperature field variations were more significant.

(2) Effect of initial temperature on the temperature field at different depths of the pavement

**Figure 13:**
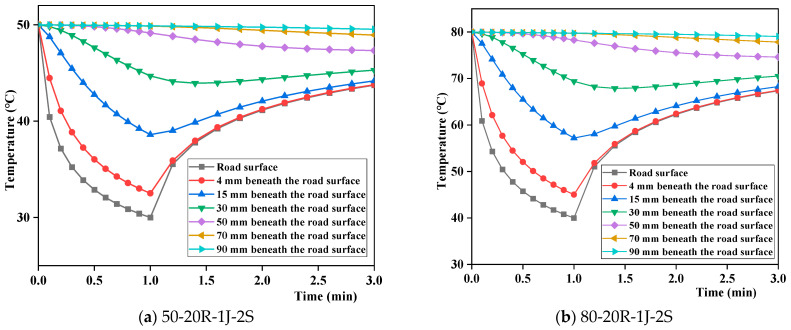
Temperature fields at different depths of the pavement with different initial temperatures.

Because the initial temperature has the same effect on the temperature at different pavement depths, only the temperature fields at different depths beneath the road surface and the initial temperatures of 50 °C and 80 °C are presented (Figure 13). It can be observed that the pavement temperature field changes along the depth direction were similar at different initial temperatures. At and beyond 30 mm from the road surface, the internal temperature of the pavement exhibited consistent variation trends with rainfall cooling and solar heating conditions. Beyond 50 mm from the road surface, the temperature inside the pavement decreases continuously and is less affected by rainfall cooling–solar heating cycles. The main reasons for this are as follows: as rainwater continuously seeps down within the pavement, its sensible heat effect [5] reduces the internal temperature of the pavement, while the range of heat conduction between rainfall events is mainly within 30 mm beneath the surface.

#### 4.3.2. Effect of Rain Temperature

(1) Effect of rain temperature on road surface temperature

**Figure 14:**
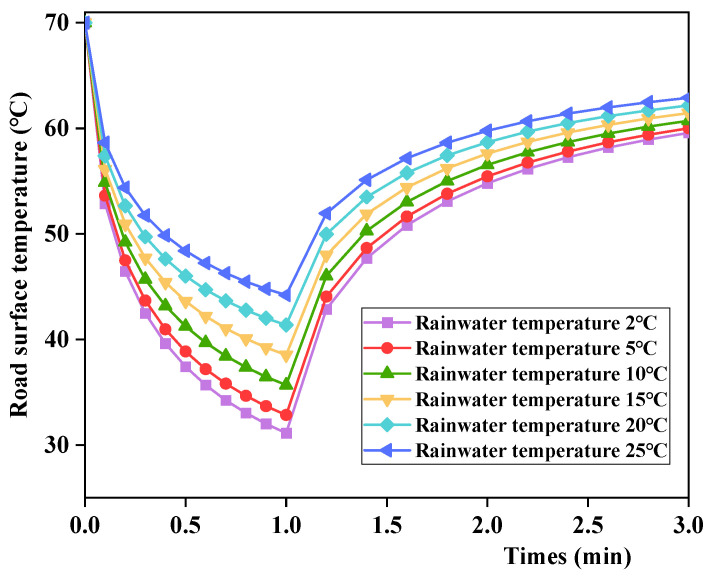
Time course of road surface temperature under different dramatic cooling–heating conditions.

As the dramatic cooling–heating cycles mainly simulate high temperatures and brief rainfall, the difference between the rain temperature and pavement temperature significantly impacts the pavement temperature field. A review of the relevant literature [30] showed that the rain temperatures in the southeastern coastal area of China were 2.6 °C to 23.1 °C. Therefore, the rain temperatures in the simulation test were set to 2 °C, 5 °C, 10 °C, 15 °C, 20 °C, and 25 °C. The test results are shown in Figure 14.

**Figure 15:**
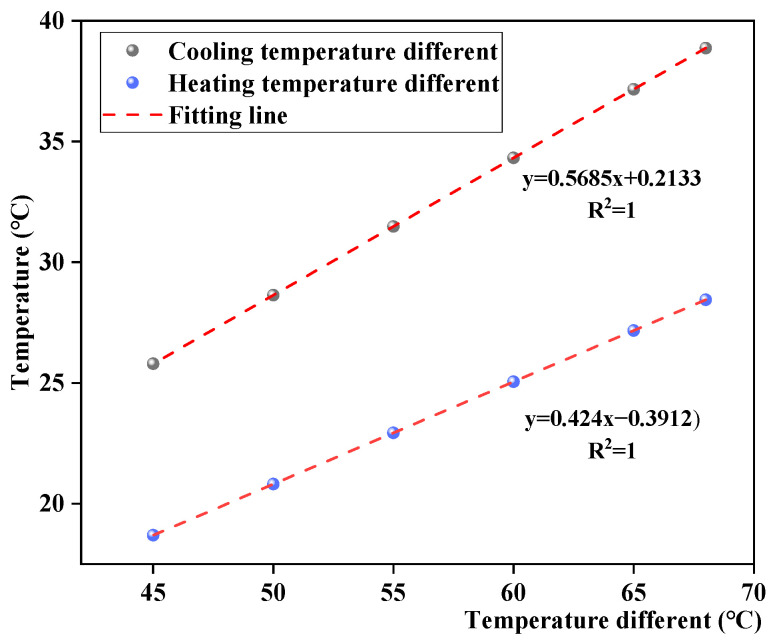
Relationship between dramatic temperature difference and pavement temperature field.

According to Figure 14, the changes in road surface temperature were greater with a lower rain temperature, that is, a greater temperature difference. With the same initial temperature of the asphalt mixture, the pavement temperature variation patterns remain unchanged under different rain temperatures, first decreasing and then increasing. Moreover, the pavement cooling rate was higher, whereas the heating rate was lower under a lower rain temperature. Further analysis found that different dramatic cooling–heating conditions have a linear relationship with the pavement temperature difference, as shown in Figure 15, and the cooling rate was larger than the heating rate.

(2) Effect of rain temperature on temperature field at different pavement depths

**Figure 16:**
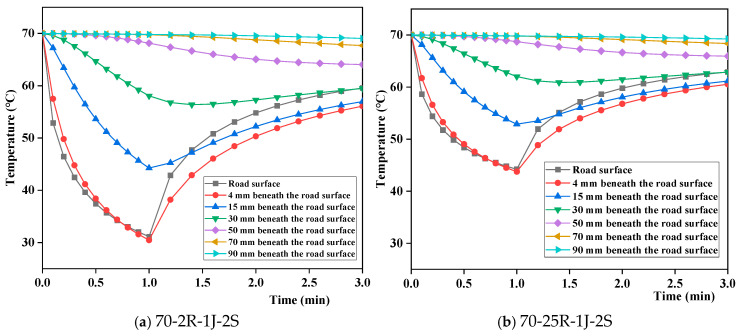
Temperature fields at different depths from the road surface at dramatic temperature differences of 68 °C and 45 °C.

Figure 16 shows the temperature fields at different depths beneath the road surface under dramatic temperature differences of 68 °C and 45 °C. With different temperature differences, the temperature fields at different depths from the road surface exhibit the same variation patterns. Below the road surface, the temperature changes were smaller. Beyond 50 mm from the road surface, the temperature decreased continuously and was unaffected by the cooling–heating cycles. Therefore, rainwater continues to seep down the road surface after the rain, and the heat from solar radiation cannot be transmitted to the lower layer over a short time, leading to a continuous temperature drop in the lower layer. The time to reach the maximum temperature difference at different depths from the road surface exhibited a hysteresis, which increased with depth.

#### 4.3.3. Effect of Rain Duration

**Figure 17:**
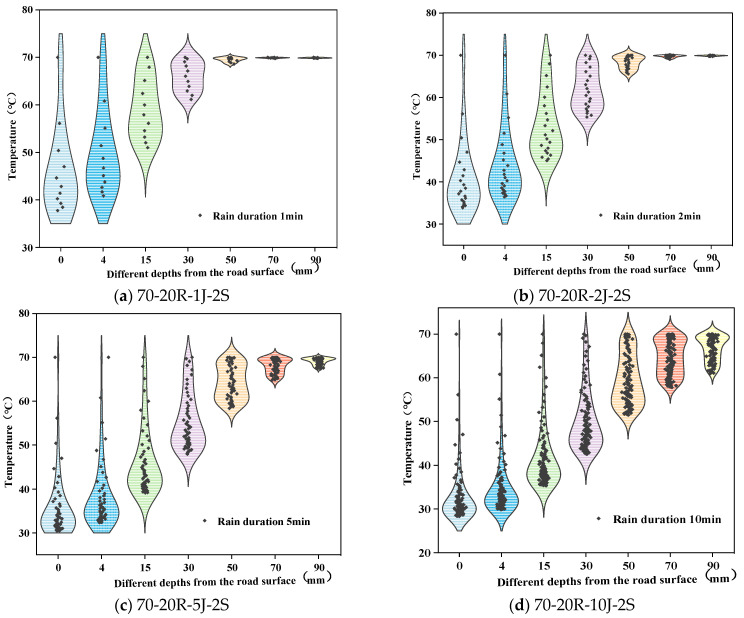
Pavement temperature field changes at different rain durations.

The impact on the pavement temperature field was greater with longer rain durations, that is, greater rain intensities. The rain durations selected in this study were 1 min, 2 min, 5 min, and 10 min, respectively, and the effect of rain duration on the pavement temperature field was analyzed. The results are shown in Figure 17.

**Figure 18:**
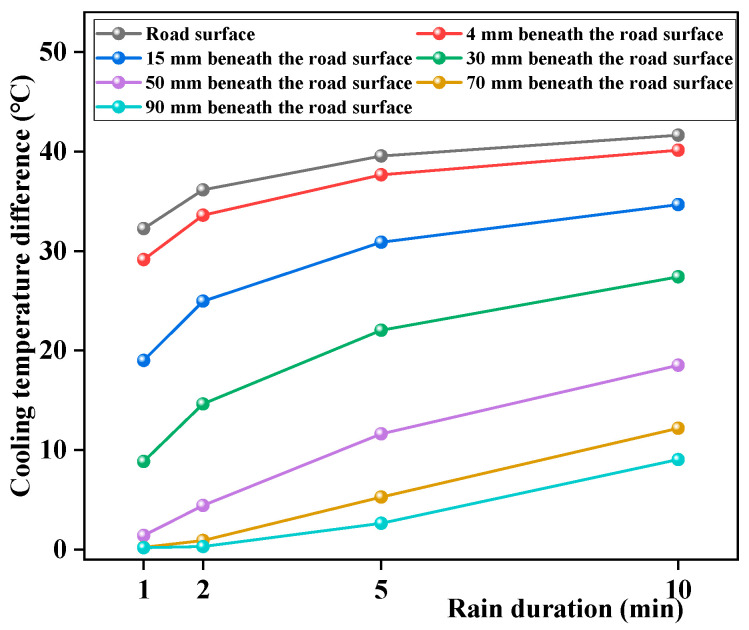
Changes in the temperature difference during rainfall with the rain intensity.

According to Figure 17, the sensible heat effect of rainwater reaches deeper with greater rain durations. At 50 mm from the road surface, the temperature decrement is 1.4 °C, 4.4 °C, 11.6 °C, and 18.5 °C, respectively, with the increase in rain duration. Thus, the effect of rainwater on the pavement temperature field reaches deeper with longer rain durations, i.e., greater rain intensities. Further analysis showed that the cooling temperature difference increased with the rain duration, and the amplitudes of these increases tended to stabilize, as shown in Figure 18. Thus, the pavement temperature field changed significantly during the early stages of rainfall. The main reason for this is that as the rain duration increases, the pavement temperature becomes increasingly closer to the rain temperature, and the heat exchange rate between them decreases.

#### 4.3.4. Effect of the Number of Cycles

**Figure 19:**
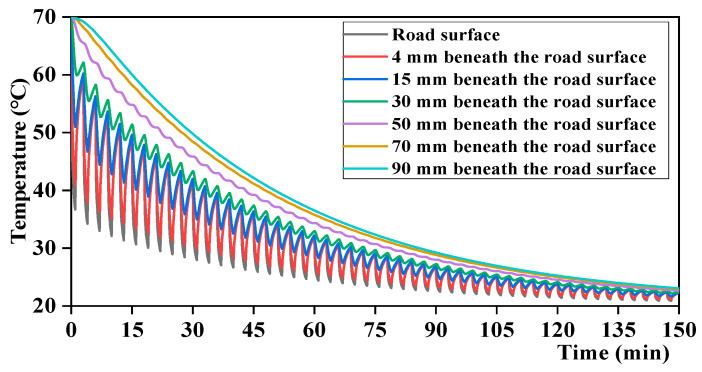
Temperature–time relationship at different depths from the road surface under 50 cycles.

The number of cooling–heating cycles was set to 50 to analyze its effect on the temperature field of the asphalt pavement. The simulation results are shown in Figure 19.

**Figure 20:**
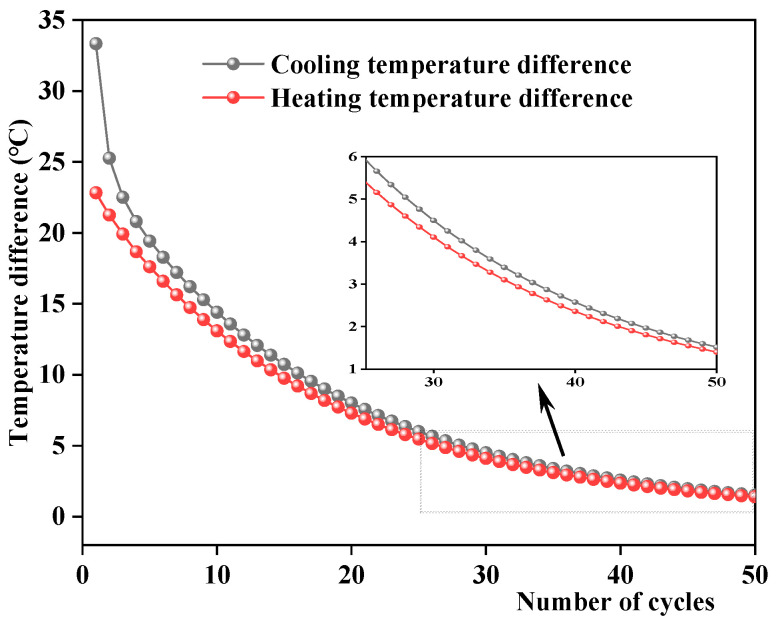
Relationship between the surface temperature difference and the number of cycles.

According to Figure 19, the pavement temperature gradually decreased as the number of cycles increased. The temperature field within 30 mm from the pavement surface exhibits a cyclic variation pattern of first decreasing and then increasing under the cooling–heating cycles, consistent with the temperature changes under different cycles measured in the laboratory. Exceeding 50 mm beneath the road surface, the pavement temperature decreases continuously. As the rain time exceeded 90 min, the variation trend of the entire pavement temperature field stabilized, indicating smaller changes in the pavement temperature field under a greater number of cooling–heating cycles. The relationships between the number of cycles, road surface cooling temperature difference, and heating temperature difference are analyzed, and the results are shown in Figure 20. The cooling and heating temperature differences of the road surface decreased with an increase in the number of cycles. In addition, as the number of cycles exceeded 30, the rate of temperature change decreased, and the cooling and heating temperature differences were basically equal. This is mainly because the temperature difference between the asphalt mixture and rain is greater with a smaller number of cycles, and the stronger heat transfer through convection increases the range of pavement temperature changes.

#### 4.3.5. Correlation Analysis of Each Influencing Factor

**Figure 21:**
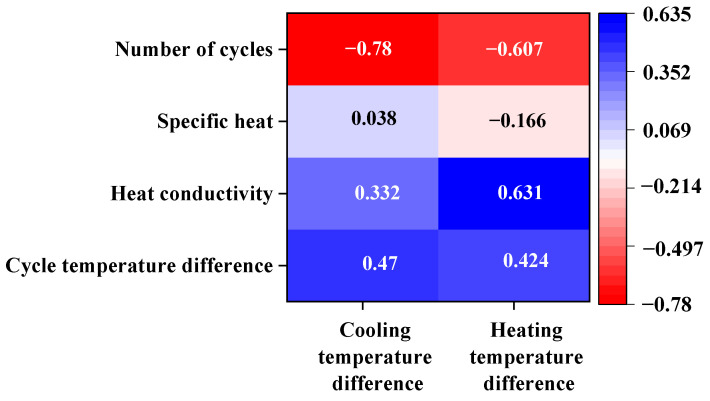
Pearson correlation analysis of various influencing factors under dramatic cooling–heating cycles.

Many factors affect the pavement temperature field under dramatic cooling–heating cycles. To quantify the weight of each influencing factor of the temperature field, Pearson correlation analysis was performed using SPSS, with the difference between the initial temperature of the asphalt mixture and the rain temperature, heat conductivity, specific heat, and the number of cycles as the contrast variables. The pavement temperature field evolution was characterized by the cooling temperature difference and heating temperature difference. The results are shown in Figure 21 and Table 8.

According to Figure 21 and Table 8, the significance levels of the relationship between the cooling temperature difference and the cycle temperature difference and the number of cycles are below 0.05, indicating significant correlations. The cooling temperature difference increased with a smaller number of cycles and a greater temperature difference. The correlation coefficients of the heating temperature difference with the cycle temperature difference, heat conductivity, and number of cycles were 0.424, 0.631, and −0.607, respectively, indicating significant correlations. The correlation coefficients were ranked as follows: heat conductivity > number of cycles > cycle temperature difference > specific heat. Thus, the heat conductivity and number of cycles have a greater impact on the heating temperature difference. Under a greater number of cooling–heating cycles, the post-rain heating rate of the pavement with high heat conductivity is greater, as is the heating temperature difference of the pavement.

## 5. Conclusions

This study employed theoretical calculations, numerical simulations, and laboratory measurements to investigate the gradient characteristics of the pavement temperature field under dramatic cooling–heating cycles. Using single variable sensitivity analysis, the effects of various external environmental factors on the pavement temperature field changes were revealed. The main conclusions are as follows.

(1) The variation characteristics of the pavement temperature field under dramatic cooling–heating cycles were studied based on a finite element model and theory. A finite element model was constructed and verified based on the laboratory data. The temperature data predicted by the finite element model exhibited good consistency with the measured data.
(2) The sensitivity of the pavement temperature field to various factors was studied, such as the initial temperature of the asphalt mixture, rain temperature, rain duration, and number of cycles. The effects of various factors on road surface temperature and internal temperature field were evaluated.
(3) The changes in the road surface temperature were greater with a higher initial temperature of the asphalt mixture and lower rain temperature. The temperature field within 30 mm from the pavement surface changed significantly, showing a pattern of first decreasing and then increasing. The rain duration has a linear relationship with the cooling temperature decrement, but the variation trend slows, and the sensible heat effect of the rain increases with the increase in rain duration. The pavement temperature decreased with an increase in the number of cycles, and the variation in the pavement temperature field was small as the number of cycles exceeded 30.
(4) Pearson correlation analysis results showed that the cycle temperature difference and the number of cycles significantly affected the cooling and heating temperature differences.

In this study, we analyzed the temperature field characteristics of pavements under the action of large wet temperature cycles with the help of indoor tests and finite element models, which build a theoretical foundation for the design and maintenance of pavements in areas with high temperatures and frequent rainfall. Next, we will conduct research on the following two aspects: first, the study of the evolution of the rheological properties of asphalt in the depth range affected by the change in the temperature field; second, the prediction of the fatigue life of asphalt in the depth range affected by the change in the temperature field based on the evolution of the law, to further determine the maintenance cycle of the pavement.

## Figures and Tables

**Table 1:** Technical specifications for the rubber-modified asphalt.

Test Items	Unit	Technical Requirements	Test Results	Test Method [21]
180 °C rotational viscosity	Pa·s	1.5~5.0	3.0	T0625
Penetration (25 °C, 100 g, 5 s)	0.1 mm	30~60	41	T0604
Softening point	℃	≥65	72	T0606
Elastic recovery	%	≥75	82	T0662
Ductility (5 °C, 1 cm/min)	cm	≥5	8	T0605

**Table 2:** Technical specifications for the SBS-modified asphalt.

Test Items	Unit	Technical Requirements	Test Results	Test Method [22]
Penetration (25 °C, 100 g, 5 s)	0.1 mm	40–60	52.1	T0604
Softening point	°C	≥60	75.5	T0606
Ductility (5 °C, 1 cm/min)	cm	≥20	26.1	T0605

**Table 3:** Grading designs for different asphalt mixtures.

Mixture Type	Sieve Size (mm) Percent Passing/%											
	26.5	19	16	13.2	9.5	4.75	2.36	1.18	0.6	0.3	0.15	0.075
AC-13	-	-	100	87.46	68.26	48.94	29.05	20.31	14.12	10.09	7.64	5.27
AC-20	100	95	83	70	59	39	26	20	16	13	7	4.4

**Table 4:** Test results of each technical index of the aggregate.

Aggregate	Test Item	Unit	Test Results	Technical Requirements	Test Method
Coarse aggregate	Apparent relative density	-	2.720	≥2.50	T 0304
	Bulk specific gravity	-	2.678	-	T 0304
	Water absorption	%	0.59	≤3.0	T 0304
	Flakiness and elongation index	%	13.4	≤15	T 0312
	Los Angeles abrasion value	%	12.5	≤30	T 0317
	Crushing value	%	26.4	≤28	T 0316
	Soft rock content	%	2.7	≤5	T 0320
Fine aggregate	Apparent relative density	-	2.690	≥2.50	T 0328
	Sand equivalent	%	70	≥60	T 0334
	Angularity	s	32.1	≥30	T 0345
	Methylene blue value	g/kg	2	≤25	T 0349

**Table 5:** Technical properties of the minerals.

Performance Index	Apparent Relative Density	Moisture Content (%)	Appearance	Particle Size Distribution (%)		
				<0.6 mm	<0.15 mm	<0.075 mm
Test value	2.63	0.93	No agglomeration	100	97.5	84.2
Technical requirements	≥2.50	≤1	No agglomeration	100	90~100	75~100

**Table 6:** Error indicators of the simulated and measured temperatures at different depths beneath the pavement surface.

Different Depths from the Road Surface(mm)	Maximum Deviation (°C)	Mean Absolute Error (MAE)	Root Mean Square Error (RMSE)
4	4.5	2.64	3.01
15	10.1	5.08	6.02
30	5.9	3.71	4.16
50	2.7	1.16	1.52
70	1.1	0.32	0.47
90	0.5	0.16	0.21

**Table 7:** Reference fixed values of various influencing factors.

Factor	Initial Temperature of the Asphalt Mixture (°C)	Rain Temperature (°C)	Rain Duration (min)	Heating Time After RAIN (min)	Number of Cooling–Heating Cycles
Reference fixed value	70	20	1	2	1

**Table 8:** Correlation analysis of influencing factors of cooling temperature decrement and heating temperature increment.

Pearson Correlation Analysis	Cycle Temperature Difference	Heat Conductivity	Specific Heat	Number of Cycles	Temperature Decrement	Temperature Increment
Temperature decrement	0.470	0.332	0.038	−0.780 **	1	0.865
	0.012 *	0.084	0.849	0.000 **	0.000 **	0.000 **
Temperature increment	0.424 *	0.631 **	−0.166	−0.607 **	0.865	1
	0.024 *	0.000 **	0.399	0.001 **	0.000 **	0.000 **

* Indicates a significant correlation at the <0.05 level (bilateral); ** indicates an extremely significant correlation at the <0.01 level (bilateral).

## Data Availability

All data are presented in the main text.

## References

[1] Achebe J., Oyediji O., Saari R.K., Tighe S., Nasir F. (2021). Incorporating Flood Hazards into Pavement Sustainability Assessment. Transp. Res. Rec..

[2] Gudipudi P.P., Underwood B.S., Zalghout A. (2017). Impact of climate change on pavement structural performance in the United States. Transp. Res. Part D Transp. Environ..

[3] Ren H., Qian Z., Huang W., Chen T., Cao H., Liu Y. (2024). Coupled effects of UV radiation and freeze–thaw cycles on the fracture behavior of asphalt concrete. Theor. Appl. Fract. Mech..

[4] Zhang L., Xing C., Gao F., Li T., Tan Y. (2016). Using DSR and MSCR tests to characterize high temperature performance of different rubber modified asphalt. Constr. Build. Mater..

[5] Zhao Y., Chen M., Wu S., Jiang Q., Xu H., Zhao Z., Lv Y. (2022). Effects of waterborne polyurethane on storage stability, rheological properties, and VOCs emission of crumb rubber modified asphalt. J. Clean. Prod..

[6] Liao J., Jiang W., Zhang X. (2017). Research on Temperature Field and Mechanical Characteristics of Full-time Domain of Asphalt Pavement. Highw. Eng..

[7] Shao L., Lian X., Tang L. (2015). Cause Analysis and Treatment Countermeasures of Early Damage of Asphalt Pavement of Bailuo Expressway in Guangxi. J. China Foreign Highw..

[8] Wang Z., Shi Y., Luo S., Chen J., Li L., Dan H. (2012). Cause analysis of early damage of typical asphalt pavement structure in Hainan Province. J. Railw. Sci. Eng..

[9] Mohseni A. (1998). LTPP Seasonal Asphalt Concrete (AC) Pavement Temperature Models.

[10] Chung Y., Shin H.C. (2015). Local Calibration of EICM Using Measured Temperature Gradients and Numerical Analysis. Int. J. Pavement Res. Technol..

[11] Chao J., Zhang J. (2018). Prediction Model for Asphalt Pavement Temperature in High-Temperature Season in Beijing. Adv. Civ. Eng..

[12] Zhao X., Shen A., Ma B. (2018). Temperature Adaptability of Asphalt Pavement to High Temperatures and Significant Temperature Differences. Adv. Mater. Sci. Eng..

[13] Zhao X., Shen A., Ma B. (2018). Temperature response of asphalt pavement to low temperatures and large temperature differences. Int. J. Pavement Eng..

[14] Li Y., Liu L., Sun L. (2018). Temperature predictions for asphalt pavement with thick asphalt layer. Constr. Build. Materials..

[15] Luo T., Tao Q., Wu J. (2015). Analysis of Temperature Field Characteristics of Asphalt Concrete Pavement Structure Based on Measured Data. J. Highw. Transp. Res. Dev..

[16] Zhang Z., Shao J., Zhao Q., Shi J., Yang X. (2022). Multi-factor prediction of permanent deformation of asphalt pavement at continuous variable temperature. J. Jiangsu Univ..

[17] Fan S., Zhu H., Lei L., Pan Y. (2023). Modeling temperature profile of asphalt mixture in semi-rigid pavement, Numerical Heat Transfer. Part A Appl..

[18] Knott J.F., Sias J.E., Dave E.V., Jacobs J.M. (2019). Seasonal and Long-Term Changes to Pavement Life Caused by Rising Temperatures from Climate Change. Transp. Res. Rec. J. Transp. Res. Board.

[19] Lu D., Tighe S.L., Xie W. (2020). Impact of flood hazards on pavement performance. Int. J. Pavement Eng..

[20] Fu J., Liu Z., Zuo X., Lai Z., Wang X., Ding Q. (2020). Influence of Regional Climate Change on Summer Temperature Effect of Asphalt Pavement. J. Chong Qing Jiao Tong Univ..

[21] (2014). Technical Specification for Construction of Asphalt Rubber Pavement.

[22] (2011). Standard Test Methods for Bitumen and Bituminous Mixtures for Highway Engineering.

[23] Qian G., He Z., Yu H., Gong X., Sun J. (2020). Research on the affecting factors and characteristic of asphalt mixture temperature field during compaction. Constr. Build. Mater..

[24] (2011). Asphalt Mixture Specimen Preparation Method (Compaction Method).

[25] Zhang J. (2014). Research of Aging Characteristic of SBSModified Asphalt under Coupling Condition of Heat and Light. Doctoral Dissertation.

[26] Sha Q. (1999). Semi-Rigid Base Asphalt Pavement of High-Grade Highway.

[27] Wang C., Zhang X., Chen X. (2007). The Change of Asphalt Pavement Temperature Field When Temperature Decreasing Suddenly. Cent. South Highw. Eng..

[28] Zhang K. (2019). Study on Lateral Crack Propagation Characteristics and Fatigue Life of Asphalt Pavement in Large Temperature Difference Area. Master’s Thesis.

[29] Zhang C., Yu H., Zhu X., Yao D., Peng X., Fan X. (2024). Unified characterization of rubber asphalt mixture strength under different stress loading paths. J. Mater. Civ. Eng..

[30] Feng X., Zhang Y., Xu G., He C., Huang C. (2020). Rainwater Temperature Prediction and Typical Urban Surface Energy Characteristics During Rainfall in the Hot-Humid Region. Build. Sci..

